# Label-Free Proteomics of the Fetal Pancreas Identifies Deficits in the Peroxisome in Rats with Intrauterine Growth Restriction

**DOI:** 10.1155/2019/1520753

**Published:** 2019-11-03

**Authors:** Xiaomei Liu, Yanyan Guo, Jun Wang, Linlin Gao, Caixia Liu

**Affiliations:** ^1^Key Laboratory of Maternal-Fetal Medicine of Liaoning Province, Department of Obstetrics and Gynecology, Shengjing Hospital of China Medical University, Shenyang 110004, China; ^2^Department of Obstetrics and Gynecology, Benxi Central Hospital of China Medical University, Benxi 117022, China; ^3^Medical Research Center, Shengjing Hospital, China Medical University, Shenyang 110004, China

## Abstract

**Aim:**

The objective of the present study was to identify differentially expressed proteins (DEPs) in the pancreas of a fetus with intrauterine growth restriction (IUGR) and to investigate the molecular mechanisms leading to adulthood diabetes in IUGR.

**Methods:**

The IUGR rat model was induced by maternal protein malnutrition. The fetal pancreas was collected at embryonic day 20 (E20). Protein was extracted, pooled, and subjected to label-free quantitative proteomic analysis. Bioinformatics analysis (GO and IPA) was performed to define the pathways and networks associated with DEPs. LC-MS results were confirmed by western blotting and/or quantitative PCR (q-PCR). The principal parameters of oxidative stress-superoxide dismutase (Sod) were determined in blood samples of fetal rats.

**Results:**

A total of 57 DEPs (27 upregulated, 30 downregulated) were identified with a 1.5-fold change threshold and a *p* value ≤ 0.05 between the IUGR and the control pancreas. Bioinformatics analysis revealed that these proteins play important roles in peroxisome biogenesis and fission, fatty acid beta-oxidation (FAO), mitotic cell cycle, and histone modification. The peroxin Pex14 was downregulated in the IUGR pancreas as confirmed by western blotting and q-PCR. Pmp70, a peroxisomal membrane protein involved in the transport of fatty acids, was upregulated. Hsd17b4 and Acox1/2, which catalyze different steps of peroxisomal FAO, were dysregulated. Sod plasma concentrations in the IUGR fetus were higher than those in the control, suggesting partial compensation for oxidative stress. Multiple DEPs were related to the regulation of the cell cycle, including reduced Cdk1, Mcm2, and Brd4. The histone acetylation regulators Hdac1/2 were downregulated, whereas Sirt1/3 and acetylated H3K56 were increased in the IUGR fetal pancreas.

**Conclusion:**

The present study identified DEPs in the fetal pancreas of IUGR rats by proteomic analysis. Downregulation of pancreas peroxins and dysregulation of enzymes involved in peroxisomal FAO may impair the biogenesis and function of the peroxisome and may underlie the development of T2 diabetes mellitus in adult IUGR rats. Disorders of cell cycle regulators may induce cell division arrest and lead to smaller islets. The present data provide new insight into the role of the peroxisome in the development of the pancreas and may be valuable in furthering our understanding of the pathogenesis of IUGR-induced diabetes.

## 1. Introduction

Intrauterine growth restriction (IUGR) indicates that the fetus failed to achieve its full growth potential. Decreased fetal growth rates reflect a temporary adaptation to the deficient intrauterine environment but may lead to malfunction of organ systems later in life. Epidemiological studies show that IUGR increases the susceptibility to type 2 diabetes mellitus (T2DM) and impaired glucose tolerance [1, 2]. Experimental models support a link between an adverse intrauterine environment and the development of diabetes [3, 4]. However, the mechanisms underlying the effect of IUGR on the fetal pancreas remain to be elucidated.

Existing reports about the impact of IUGR on the pancreas mainly focus on islet and *β* cells. The conclusions reached in these studies are controversial, which may be attributed to the use of different animal models and time points in various studies. In most reports, IUGR is associated with decreased *β*-cell mass and insulin secretion [5]. Rats with IUGR caused by a maternal low-protein diet show disordered acini at embryo day 14, loosely arranged pancreatic tissue at E19, and decreased islet areas and quantities in the neonate [6]. Hyperthermia-induced IUGR fetal sheep are characterized by low glucose-stimulated insulin secretion (GSIS), small islets with low amounts of *β* cells, and impaired pancreatic vascularity [7]. Increased GSIS and insulin sensitivity are observed in young lambs with hyperthermia-induced IUGR [8]. In addition to the decreased *β*-cell mass, the IUGR fetus shows decreased *α*-cell mass, and the functions of other cells may be affected as well [7].

Perturbations in the intrauterine environment can dysregulate gene expression and function in susceptible cells and affect the development of the pancreas leading to adult diabetes [9–11]. The objective of the present study was to elucidate the mechanisms underlying the impairment in the development and metabolism of the pancreas of fetuses with IUGR using large-scale proteomic analysis. The impact of IUGR on the proteomic profile of the fetal pancreas was investigated using isobaric tags for relative and absolute quantization (label-free) technology coupled with liquid chromatography-tandem mass spectrometry (LC-MS/MS) to define the mechanisms underlying reduced growth, impaired metabolism, and decreased insulin secretion in the pancreas of IUGR fetuses.

## 2. Materials and Methods

### 2.1. Animals and Tissue Collection

The animal study protocols were approved by the Animal Research Committee of China Medical University (ethics approval number: 2018PS41K). Adult Wistar rats (body weight, 230–260 g) were individually housed under specific pathogen-free conditions in an environmentally controlled clean room at the Experimental Animal Center of Shengjing Hospital of China Medical University. Food and water was provided *ad libitum* throughout the study period. Female rats were randomly divided into two groups after mating with male rats. Animals in the under-nourished group were fed an isocaloric low-protein diet (7% protein) from day 0 of pregnancy until the delivery of pups as described previously [12], whereas control animals were maintained on a conventional diet (23% protein) during gestation. At 20 days of gestation (term, 21 days), pups were delivered by caesarian section and decapitated. Fetal blood was pooled (three or more) from the control or IUGR fetuses in a litter to quantify plasma Sod levels using the Abbott Architect ci16200 automatic biochemical-immune analyzer. The pancreas was removed immediately, pooled (three), frozen in liquid nitrogen, and stored at –80°C until assays were performed. IUGR refers to a fetus (E20) with a body weight of at least two standard deviations lower than the average body weight of the normal fetus. The remaining pups were nourished by their mothers until weaning at 3 weeks postnatal and fed under normal conditions until 12 weeks (12 W). Eight pups from three dams in each group were killed under ether anesthesia at 12 W; the pancreases were excised, rinsed in saline, and frozen at −80°C until analysis. To avoid any interference due to sex and hormonal differences, the analyses at 12 W were performed on male pups only.

### 2.2. Preparation of Tissues for Proteomic Analysis

**Figure 1:**
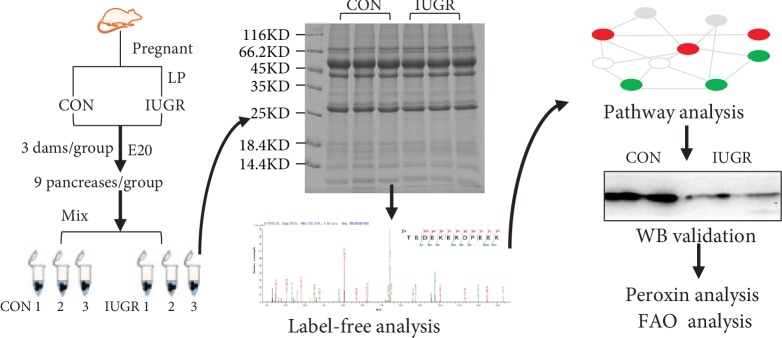
Outline of the experimental workflow.

Figure 1 shows the workflow of this study. Briefly, 6 dams (3 controls, 3 IUGR) were used for label-free quantification (LFQ). Three fetal pancreases from a single dam were pooled together as one sample. The pancreases of 9 control fetuses from three dams were pooled into 3 samples as control1, control2, and control3, and 9 IUGRs from three dams were pooled into 3 samples as IUGR1, IUGR2, and IUGR3. All pancreatic tissue was homogenized in SDT buffer (4% sodium dodecyl sulfate (SDS), 1 mM dithiothreitol (DTT), 150 mM Tris-HCl, pH 8.0). The homogenate was sonicated on ice after boiling for 15 min. After centrifugation at 14000g for 40 min, the supernatant was filtered through 0.22 *μ*m filters. The filtrate was quantified using the BCA method. Aliquots containing 20 *μ*g of protein were separated by SDS-PAGE, and protein bands were visualized by Coomassie Blue R-250 staining to observe the quality of the extracted protein. Samples were stored at -80°C.

### 2.3. Filter-Aided Sample Preparation and Digestion

Aliquots containing 200 *μ*g of protein for each sample were incorporated into 30 *μ*l SDT buffer (4% SDS, 100 mM DTT, and 150 mM Tris-HCl pH 8.0). The detergent, DTT, and other low-molecular-weight components were removed using UA buffer (8 M urea, 150 mM Tris-HCl pH 8.0) by repeated ultrafiltration (Microcon units, 10 kD). Then, 100 *μ*l iodoacetamide (100 mM IAA in UA buffer) was added to block reduced cysteine residues, and the samples were incubated for 30 min in the dark. The filters were washed with 100 *μ*l UA buffer three times followed by 100 *μ*l of 25 mM NH_4_HCO_3_ buffer twice. Finally, the protein suspensions were digested with 4 *μ*g trypsin (Promega) in 40 *μ*l of 25 mM NH_4_HCO_3_ buffer overnight at 37°C, and the resulting peptides were collected as a filtrate. The peptides of each sample were desalted on C18 cartridges (Empore™ SPE Cartridges C18 (standard density), bed I.D. 7 mm, volume 3 ml, Sigma), concentrated by vacuum centrifugation, and reconstituted in 40 *μ*l of 0.1% (*v*/*v*) formic acid. The peptide content was estimated by UV light spectral density at 280 nm using an extinction coefficient of 1.1 of 0.1% (g/l) solution that was calculated based on the frequency of tryptophan and tyrosine in vertebrate proteins.

### 2.4. Mass Spectrometry

LC-MS/MS analysis was performed on a Q Exactive mass spectrometer (Thermo Scientific) coupled to Easy nLC (Proxeon Biosystems) for 120 min. Positive ion mode was chosen to operate the mass spectrometer. MS data were acquired using a data-dependent top 10 method dynamically choosing the most abundant precursor ions from the survey scan (300–1800 m/z) for HCD fragmentation. The automatic gain control target was set to 3e6, and the maximum injection time was set to 10 ms. The dynamic exclusion duration was 40.0 s. Survey scans were acquired at a resolution of 70,000 at *m*/*z* 200, the resolution for HCD spectra was set to 17,500 at *m*/*z* 200, and the isolation width was 2 *m*/*z*. Normalized collision energy was 30 eV, and the underfill ratio, which specifies the minimum percentage of the target value likely to be reached at maximum fill time, was defined as 0.1%. The instrument was run with the peptide recognition mode enabled.

### 2.5. Data Analysis

The MS data were analyzed using MaxQuant software (version 1.5.3.17) against the UniProtKB rat database (36,079 total entries, downloaded 09/21/17) to produce a list of protein groups and their corresponding intensities in each group. Label-free quantity comparison between the two groups was performed by DeCyder MS Differential Analysis Software.

### 2.6. Bioinformatics Analysis

Only the proteins detected in *n* ≥ 2 individual pancreatic tissues from the control or the IUGR group were selected as quantified proteins. The *p* value of the differences in proteins between the two groups was calculated using Student's *t*-test. The criterion for significant change was set to a 1.5-fold change (FC) ratio between the control and IUGR groups and a confidence value of *p* ≤ 0.05. The differentially expressed proteins were annotated using the Database for Annotation, Visualization, and Integrated Discovery (DAVID, v6.7) with the whole rat proteome as the background (http://www.uniprot.org/). Three ontologies (biological process, molecular function, and cellular component) of identified proteins were further analyzed using information from Gene Ontology (GO) (http://www.geneontology.org/). To further explore the impact of differentially expressed proteins on cell physiological processes and discover internal relations between differentially expressed proteins, GO enrichment analyses of three ontologies were performed based on Fisher's exact test, considering the whole quantified protein annotation as the background dataset. Benjamini-Hochberg correction for multiple testing was further applied to adjust derived *p* values. Only functional categories and pathways with *p* values < 0.05 were considered significant. Pathway and network analyses were performed with Ingenuity Pathway Analysis (IPA) software.

### 2.7. Western Blot Analysis

As with the LFQ sample, three fetal pancreases from a single dam were pooled together and total proteins were extracted using SDT buffer and quantified using the BCA method. Equal amounts of proteins were separated by 8%–15% SDS-PAGE and transferred onto a PVDF membrane (EMD Millipore). After transfer, the membrane was cut into several strips to detect different target proteins according to molecular weight. Membranes were probed with primary antibodies (supplement [Supplementary-material supplementary-material-1]) overnight at 4°C, followed by horseradish peroxidase- (HRP-) conjugated secondary antibodies (1 : 3,000; ZSGB-BIO, Beijing, China), and detected using Immobilon Chemiluminescent HRP substrate (Millipore) on a ChemiDoc XRS Imaging System (Bio-Rad Laboratories). Band optical density quantification was performed using GelAnalyzer software.

### 2.8. Pancreas Histology Staining

The fetal pancreas was removed and fixed in 4% paraformaldehyde for 24 h before processing using an automatic tissue processor for paraffin embedding. Sections 3 *μ*m thick were subjected to immunofluorescence (IF) staining according to standard procedures with Pex14 antibody (Supplemental [Supplementary-material supplementary-material-1]). Immunoassays without primary antibodies served as the negative control. Imaging was performed using a confocal microscope (Nikon C1, Tokyo, Japan).

### 2.9. mRNA Analysis

Total RNA was prepared from frozen pancreas tissues homogenized in liquid N2 and extracted with TRIzol (Invitrogen Corporation, Carlsbad, CA, USA) according to the manufacturer's protocol with addition of guanidine hydrochloride to denature proteins and limit the digestive effect of trypsin on RNA. The purity and concentration of total RNA were assessed using a NanoDrop spectrometer (GE Healthcare, Buckinghamshire, England), and A260/280 ratio was between 1.8 and 2.0. The integrity of RNA was checked through capillary gel electrophoresis using the QIAxcel Advanced System. RNA Integrity Score (RIS) and peak images were assessed, and only RNA with a RIS ≥ 6 were used (RIS: 6.2-8.7 for all samples). Aliquots containing 1 *μ*g of total RNA from each sample were reverse transcribed in a 40 *μ*l reaction with the M-MLV First Strand kit (C28025-032; Invitrogen Corporation). Real-time PCR was performed on an ABI 7500 Fast system (Hercules, CA, USA) with the following parameters: initial denaturing step at 95°C for 10 min, followed by 40 cycles of 95°C for 15 s and 60°C for 20s. One microliter of cDNA and SYBR Green I Master Mix (ABI Applied Science) in a 20 *μ*l reaction was used in real-time PCR. The primer sequences are listed in Supplementary [Supplementary-material supplementary-material-1]. The relative mRNA levels were calculated using the 2-*ΔΔ*Ct method after normalization against *β*-actin as a reference gene. Student's *t*-test was used to assess statistical significance.

### 2.10. Statistical Analysis

The data are presented as the mean ± standard error of the mean (SEM). The results were analyzed using Student's unpaired *t*-test with SPSS 17.0 (SPSS Inc., Chicago, IL, USA). A two-sided *p* value < 0.05 was considered significant.

## 3. Results

### 3.1. Identification of Significant Differentially Expressed Proteins (DEPs) in the Fetal Pancreas

**Figure 2:**
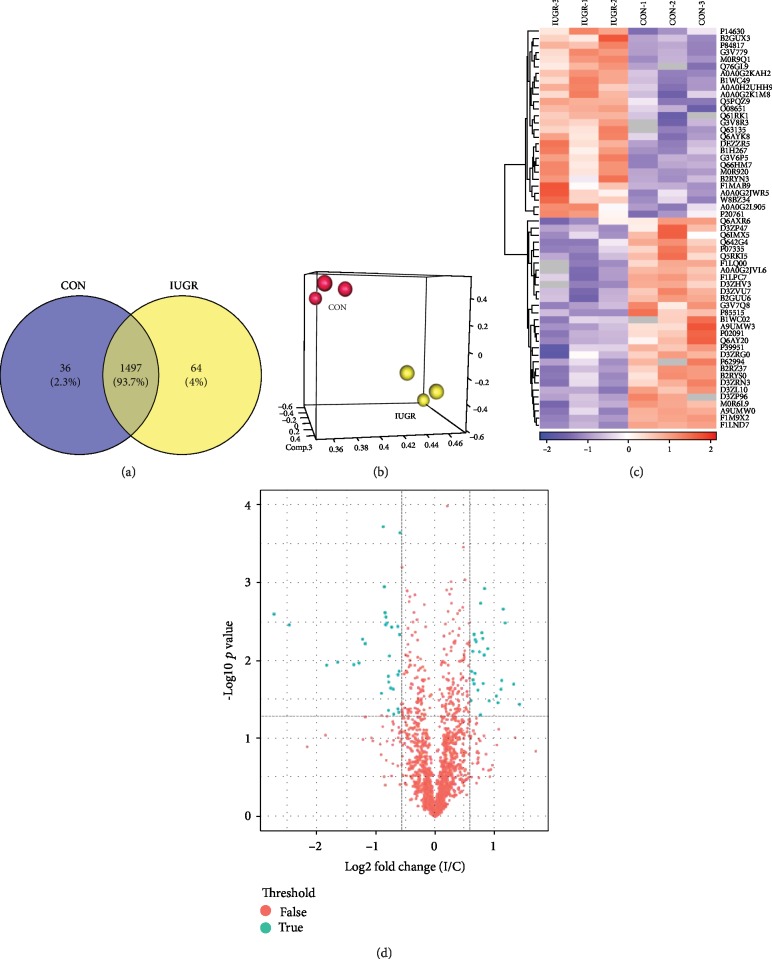
Global protein expression patterns in the rat fetal pancreas. (a) Venn diagram showing 1,497 proteins identified in both groups (overlap), 36 proteins detected only in the control group (blue), and 64 proteins detected only in the IUGR group (yellow). (b) Principal component analysis (PCA) graph showing a clear separation between control and IUGR samples. Each point in the PCA graph represents the protein profile of one biological replicate sample. (c) *K*-means clustering representation of the profiles of 57 differentially expressed proteins. The percentage variation is represented by a color scale (top right) from low (blue) to high (red). (d) Volcano plots of the 1,497 quantified proteins showing the distribution of significance and fold change of identified proteins (the logarithmic ratio of protein LFQ intensities in the IUGR/CON comparison was plotted against negative logarithmic *p* values of the *t*-test). Vertical dotted lines mark a fold change of ±150%, and horizontal dotted lines mark a *p* < 0.05.

LC-MS/MS analysis identified 10,704 peptides matching 1,597 proteins (≥one unique peptide with an FDR < 1%). Of these, 1,497 were present in both groups. Proteins with zero data detected by mass spectrometry in one of the two groups were named no/have proteins. No or Have is meant for the IUGR group. No means zero in the IUGR group; Have means zero in the control group. These proteins are defined as No or Have as follows: there are more than two values in one group, and the other group is all zero. 36 proteins were zero in the IUGR group (No) and 64 were zero in the control group (Have) (Figure 2(a)). The principal component analysis (PCA) performed based on multivariate data (Figure 2(b)) showed a clear separation between control and IUGR samples.

After applying a 1.5-fold threshold and a *p* value < 0.05, 57 differentially expressed proteins (DEPs) (27 upregulated and 30 downregulated) were identified between the IUGR fetal pancreas and the control. The significance and magnitude of expression change of DEPs between the two groups were identified using *K*-means clustering heatmaps and a volcano plot (Figures 2(c) and 2(d)).

### 3.2. Gene Ontology and Functional Enrichment Analysis

**Figure 3:**
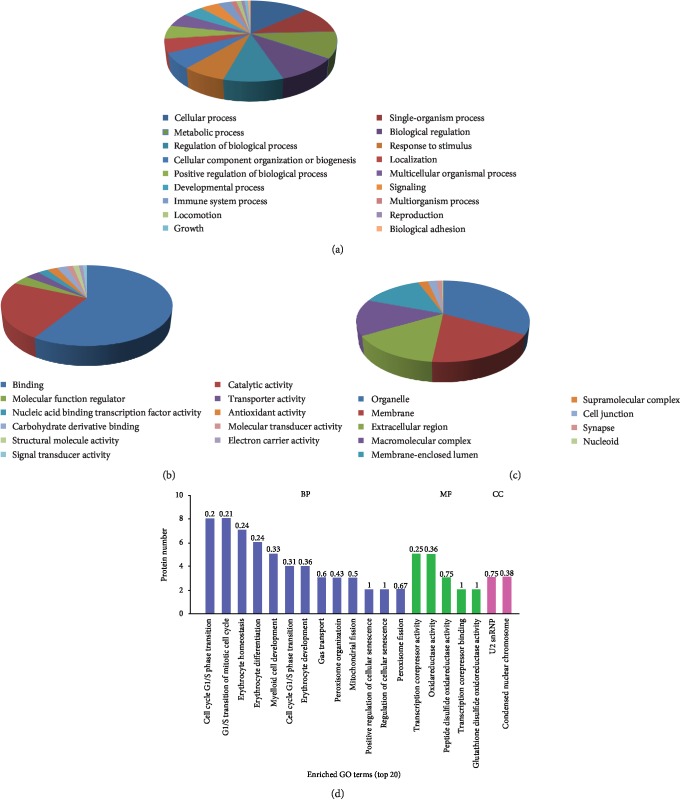
GO annotation and functional enrichment of differential proteins (including 57 expressed proteins and 100 no/have proteins).GO terms for subcellular location distribution (a), molecular functions (b), and biological process (c). (d) Enrichment analysis shows the top 20 enriched GO terms associated with differential proteins.

A total of 157differential proteins (57DEPs and 100 No/Have proteins) were annotated to 3,506 GO function entries. A Gene Ontology analysis with second-level GO terms was used to classify proteins according to their involvement in three main categories (cellular component, molecular function, and biological process). The differential proteins were mainly categorized into organelle (135 proteins), membrane (76), extracellular region (60), and macromolecular complex (59). The molecular function classification showed that most of the differential proteins were associated with protein binding (128), catalytic activity (50), and molecular function regulator [8]. The top three biological processes identified were the cellular process (135), single-organism process (112), and metabolic process (107) (Figure 3(a)–3(c)). The GO enrichment analysis defined the significantly enriched categories, including peroxisome organization and fission, regulation of cell cycle, and oxidoreductase activity (Figure 3(d)).

### 3.3. Canonical Pathways and Network Analysis

**Figure 4:**
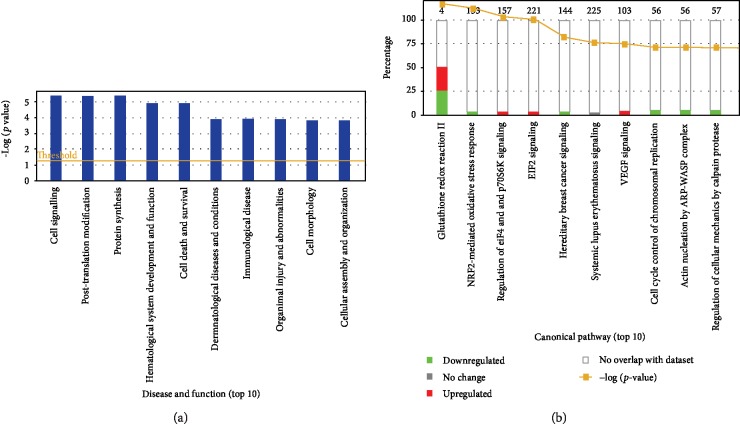
Ingenuity pathway analysis identified the top functions and canonical pathways associated with differential proteins. (a) Top 10 diseases and functions associated with DEPs in the fetal pancreas. (b) Top10 canonical pathways associated with DEPs in the fetal pancreas. Red, green, and gray indicate the percent of upregulated, downregulated, or no change proteins that matched each pathway, respectively. The orange line indicates the *p* value of the association between the reference and focus proteins for each pathway. The number on top of each pathway indicates the total number of proteins associated with the corresponding pathway.

**Figure 5:**
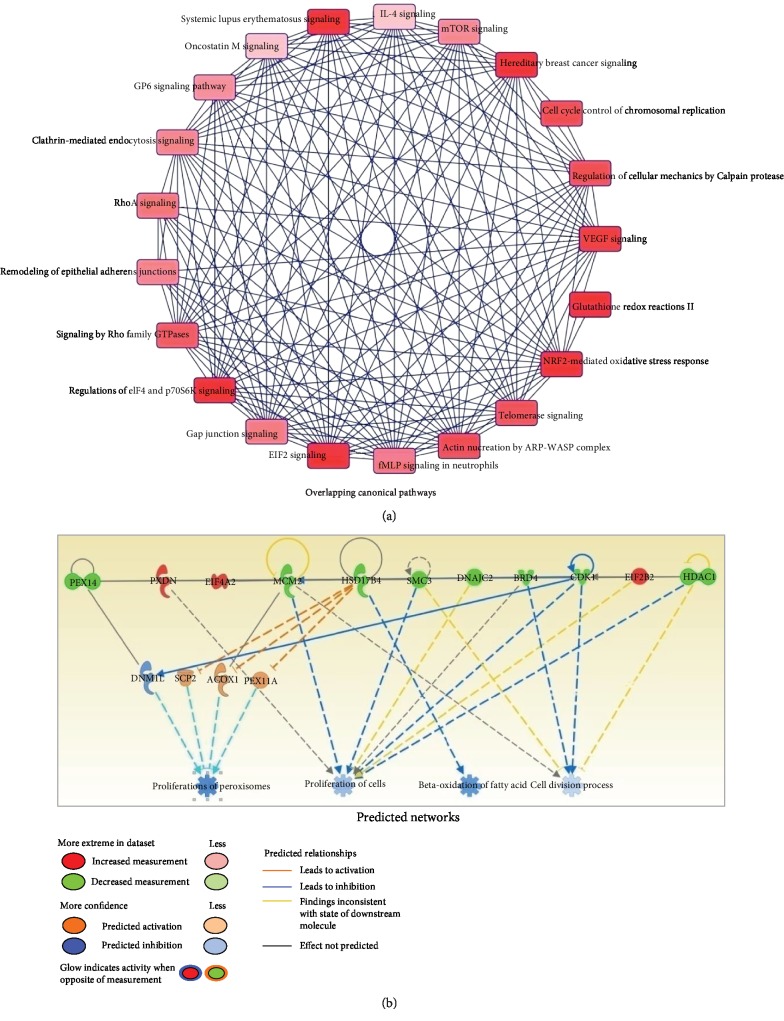
IPA identified overlapping canonical pathways and predicated networks associated with differential proteins. (a) Overlapping canonical pathways associated with differential proteins. (b) Predicted protein interaction network was generated with differential proteins associated with peroxisome and cell cycle by IPA.

The 157 differential proteins were analyzed using the IPA database to identify molecular networks and pathways potentially affected in IUGR. The top ranked diseases and functions, and canonical pathways are listed in Figure 4. These proteins were most frequently involved in cell signaling, posttranslational modification, and protein synthesis (Figure 4(a)). The top canonical pathways identified were the glutathione redox reactions II, NRF2-mediated oxidative stress response, regulation of eIF4 and p70S6K signaling, EIF2 signaling, and hereditary breast cancer signaling (Figure 4(b)). The network of functions generated using the IPA database is shown in Figure 5(a). A predicated network was further developed including the differential proteins related to the peroxisome, cell cycle, and endoplasmic reticulum stress (ERS). The network showed that the differential proteins interact to regulate peroxisome proliferation, fatty acid beta-oxidation, and cell proliferation and division (Figure 5(b)). A selection of differential proteins is listed in Table 1.

### 3.4. Investigation of Peroxisomal-Related Proteins

**Figure 6:**
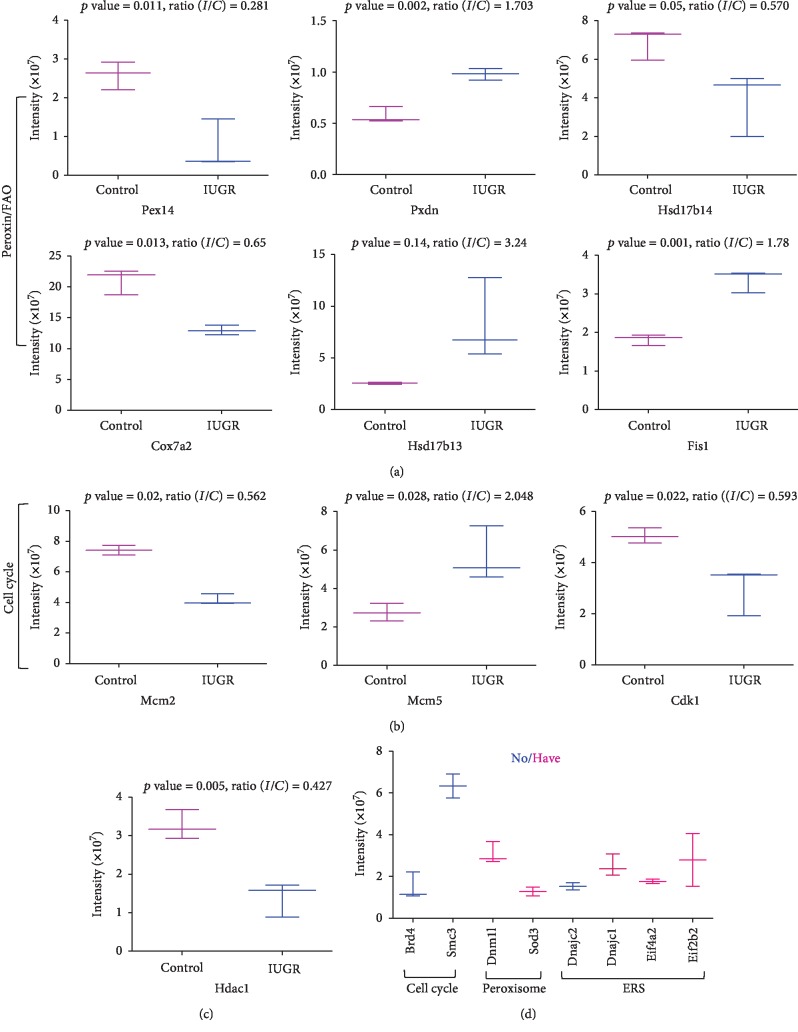
Box plot for the LFQ intensity of focus DEPs associated with the peroxisome and cell cycle in LFQ proteomic analysis. (a) Peroxisome-related factors. (b) Cell cycle regulators. (c) HDAC1. (d) Label-free analysis of focus proteins detected in control or IUGR specimens. The results were expressed as the mean ± SEM. *n* = 3 per group, ^∗^*p* < 0.05 vs. control.

**Figure 7:**
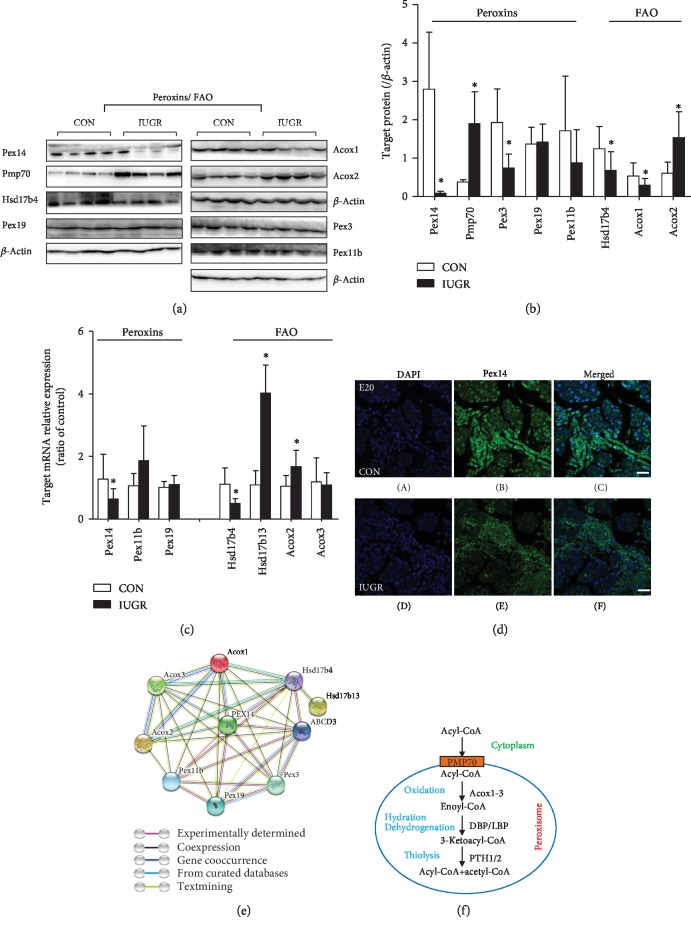
Investigation of peroxisomal-related proteins in the fetus. (a) Western blot analysis of peroxisome-related factors in the fetal pancreas. (b) Densitometry analysis results were expressed as the mean ± SEM. *n* = 5–8, ^∗^*p* < 0.05 vs. control. (c) The mRNA levels of focus proteins in the fetal pancreas were determined by quantitative RT-PCR with *β*-actin as the reference gene. (d) Representative photomicrographs of immunofluorescence analysis of Pex14 in sections of the pancreas from the control (A, B, C) and IUGR (D, E, F) (original magnification 400x). (e) The STRING network of peroxins and peroxisomal FAO with altered expression. Colored lines between the proteins indicate the various types of interaction evidence. (f) Schematic representation of the peroxisomal FAO, which consists of 4 steps catalyzed by different enzymes and leading to the formation of a chain-shortened acyl-CoA and acetyl-CoA.

Label-free data showed that there were multiple differential proteins related to the peroxisome, including Pex14, Dnm1l, Fis1, Hsd17b4, and Hsd17b13 (Figure 6). Pex14, an optional marker for the peroxisome, was dramatically downregulated in the IUGR group, whereas Dnm1l and Fis1, which are involved in peroxisomal fission, were upregulated. Hsd17b4, which catalyzes the second step of peroxisome fatty acid beta-oxidation (FAO), was downregulated in the IUGR group (Figure 6). Western blot analysis confirmed the differential expression of Pex14 and Hsd17b4 (Figures 7(a) and 7(b)). Pmp70; other peroxins Pex3, Pex19, and Pex11b; and the acyl-CoA oxidase (Acox)1/2 were not detected by LC-MS. Western blot analysis showed that the peroxisomal biogenesis-associated gene Pex3 was downregulated, whereas Pex11b showed a decreasing trend without statistical significance, which could be attributed to high intersample variation. Pex19 levels were unchanged. Pmp70, a membrane protein involved in the transport of fatty acids, was upregulated in the IUGR group. Acox1, the rate-limiting enzyme responsible for the initial step of peroxisomal FAO, was significantly downregulated, whereas Acox2 was upregulated. Consistent with the results of western blotting, fluorescence microscopy images showed decreased Pex14 fluorescence intensity in the fetal pancreas of the IUGR group (Figure 7(d)). Protein-protein interactions, including direct (physical) and indirect (functional) associations, were confirmed using the STRING database (Figure 7(e)).

The mRNA levels of selected peroxins and FAO regulators were investigated by q-PCR in the fetal pancreas (Figure 7(c)). Pex14 and Hsd17b4 were downregulated in the IUGR pancreas, whereas Hsd17b3 and Acox2 were upregulated, consistent with the western blotting or LC-MS results.

Regarding antioxidant enzymes, plasma Sod levels were higher in the IUGR fetus than in the control group (42.58 ± 13.63 vs. 74.82 ± 5.94 IU/ml, *p* < 0.05), consistent with LC-MS data. This increase could reflect an adaptive response to the peroxisome FAO defect.

**Figure 8:**
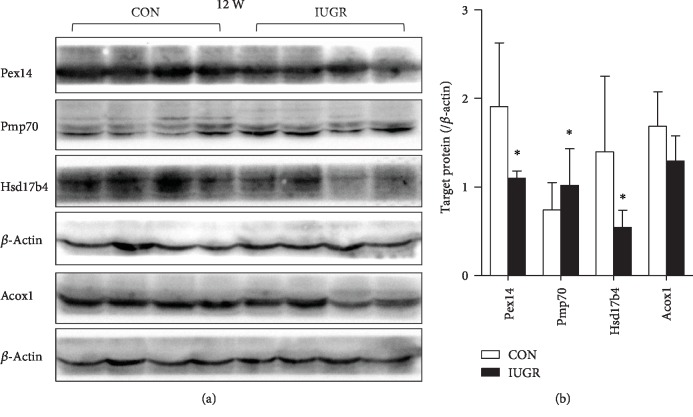
Peroxisomal-related proteins in adult offspring. (a) Western blot analysis of critical peroxisome factors in the adult pancreas. (b) Densitometry analysis results were expressed as the mean ± SEM. *n* = 6–8, ^∗^*p* < 0.05 vs. age-matched control.

We further investigated the protein level of key peroxins in the pancreas of adult offspring (12 W). Consistent with fetus results, pancreatic Pex14 and Hsd17b4 were remarkably downregulated, while Pmp70 was increased in IUGR rats. Yet, Acox1 was restored to the normal level (Figure 8).

### 3.5. Investigation of Cell Cycle Regulators

**Figure 9:**
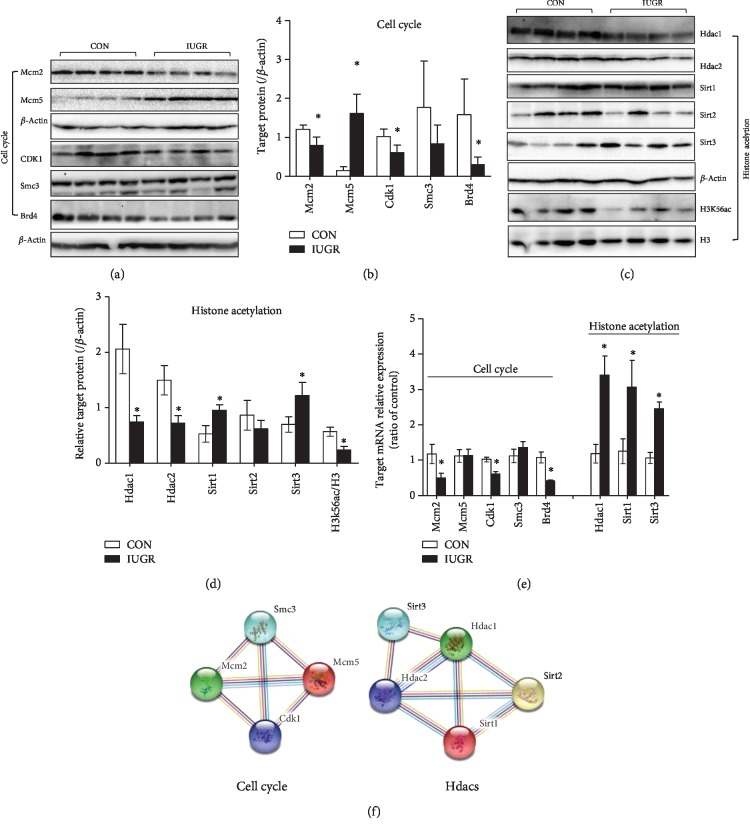
Cell cycle regulators and Hdacs. (a, b) Representative immunoblotting and densitometric quantification of cell cycle regulators in the fetal pancreas. (c, d) Representative immunoblotting and densitometry analysis of Hdacs in the fetal pancreas. Results were expressed as the mean ± SEM. *n* = 6–8, ^∗^*p* < 0.05 vs. control. (e) The mRNA levels of these proteins in the fetal pancreas were determined by quantitative RT-PCR with *β*-actin as the reference gene (*n* = 6 per group, ^∗^*p* < 0.05, vs. control). (f) The STRING networks of cell cycle regulators and Hdacs with altered expression. Colored lines between the proteins indicate the various types of interaction evidence.

LC-MS analysis indicated that there were multiple differential proteins related to cell cycle regulation, including cyclin-dependent kinase 1 (Cdk1), Mcm2/5, Smc3, and bromodomain-containing protein 4 (Brd4) (Figure 6). Western blot analysis confirmed that Cdk1, Mcm2, and Brd4 were significantly downregulated, whereas Mcm5 was upregulated in the fetal pancreas of the IUGR group compared with that of the control, consistent with the LC-MS/MS data (Figures 9(a) and 9(b)). Smc3 showed a tendency toward lower expression in the IUGR fetal pancreas(-95%, *p* = 0.066), whereas it was not detected in the IUGR fetal pancreas by LC-MS. Assessment of mRNA levels by q-PCR showed that Mcm2, Cdk1, and Brd4 were downregulated in the IUGR pancreas, whereas Mcm5 and Smc3 were unchanged (Figure 9(e)). Protein-protein interactions were confirmed using the STRING database (Figure 9(f)).

### 3.6. Investigation of Epigenetic Modulating Factors

Label-free data showed that Hdac1, a histone deacetylase, was downregulated in the IUGR fetal pancreas (Figure 6). We further investigated the histone deacetylase superfamily (Hdac1/2, Sirt1/2/3). Western blot analysis confirmed that the Hdac1 and Hdac2 proteins were markedly reduced in the IUGR group. The expression of Sirt1 and Sirt3 was higher in the IUGR fetus than in the control, and H3K56ac was remarkably downregulated in the IUGR fetal pancreas (Figures 9(c) and 9(d)). The mRNA level of Sirt1 and Sirt3 were increased in the IUGR group, consistent with the LC-MS data. However, the Hdac1 mRNA level was significantly higher in the IUGR fetus than in the control group, which was not in agreement with the LC-MS data (Figure 9(e)).

## 4. Discussion

IUGR causes permanent and progressive changes in gene expression that affect important metabolically active tissues such as the pancreas, leading to an increased risk of developingT2DM in adulthood [6, 11]. The IUGR offspring often show disordered pancreatic tissues, small islets, and decreased insulin secretion [4, 5]. Elucidating the mechanisms underlying alterations in pancreas development may help the design of therapeutic strategies and decrease the risk of diabetes in the IUGR offspring.

In the present study, the sample-pooling comparative proteomic method was used to investigate the differences in the proteomic profiles of the pancreas between the IUGR fetus and the normal fetus, which led to the identification of 157 differential proteins. Functional classification of these differential proteins showed that they play important roles in the regulation of peroxisome organization and fission, oxidoreductase activity, and mitotic cell cycle.

Peroxisomes are multifunctional organelles involved in ROS metabolism, fatty acid oxidation, ether lipid synthesis, bile acid synthesis, and cholesterol transport. Impaired peroxisomal function has been implicated in insulin resistance and diabetes [13]. Peroxisomes contain more than 30 specific peroxisomal proteins named peroxins [14]. Pex14, a central component of the peroxisomal protein translocation machinery, is a multitasking protein that not only facilitates peroxisomal protein import but is also required for peroxisome motility by serving as a membrane anchor for microtubules [15]. Pex14 mutation can cause Zellweger syndrome, which is clinically manifested by hyperbilirubinemia, hypoketotic dicarboxylic aciduria, and low plasmalogen concentration [16]. Pex14 silencing leads to defective peroxisomal biogenesis and metabolism, resulting in a partial defect in peroxisomal matrix protein import and impaired catalase import [17]. Consistent with the decreased levels of pancreatic Pex14, the peroxisomal biogenesis-associated protein Pex3 was also downregulated in the IUGR fetus. Knockdown of Pex3 leads to a reduced number of peroxisomes [18]. The ABC transporter (Abcd3/Pmp70) is an integral membrane protein involved in the transport of fatty acids across the peroxisomal membrane. Hsd17b4, a D-bifunctional protein, catalyzes the second step of peroxisomal FAO and leads to the formation of a chain-shortened acyl-CoA and acetyl-CoA [19]. It also acts as a catalyst for the formation of 3-ketoacyl-CoA intermediates from both straight-chain and 2-methyl-branched-chain fatty acids. Hsd17b4 and Pmp70 are essential for the peroxisomal oxidation of lauric and palmitic acid. The present results showed that Hsd17b4 was downregulated, whereas Pmp70 was upregulated in the IUGR pancreas from the fetus to adulthood. The effect of the opposite changes in the expression of the two critical factors on FAO in the pancreas needs to be investigated further.

Peroxisomes play a role in maintaining cellular redox homeostasis. Deficiency of peroxisomes leads to increased oxidative stress in different peroxisomal disorders [20, 21]. Peroxisomes contain more than 50 enzymes that function in the neutralization of ROS generated during FAO. Acox is the first and rate-limiting enzyme in fatty acid *β*-oxidation and also a major producer of H_2_O_2_. Three Acox proteins form homo- and heterodimers with distinct substrate preferences. The Acox1/2 heterodimer catalyzes the first step in the *β*-oxidation cycle [22]. Acox dysfunction is linked to various peroxisomal disorders [23]. In the present study, Acox1 was downregulated in the IUGR fetus, which might lead to peroxisomal FAO disorders. By contrast, Acox2 was upregulated in the IUGR group, which may represent a compensation for the function of the heterodimer. LC-MS detected high Sod3 protein expression in the IUGR fetal pancreas. Similarly, plasma total Sod activity was higher in the IUGR fetus than in the controls. This is consistent with a previous study reporting increased oxidative stress in IUGR patients and increased Sod activity in the maternal plasma [24]. Increased Sod levels suggested partial compensation for oxidative stress.

The present results showed that Fis1 and Dnm1l were upregulated in the IUGR fetal pancreas. Fis1 mediates both mitochondrial and peroxisomal fission by interacting with Dnm1l [25]. Silencing of Fis1 and Dnm1l inhibits fission and induces elongation of peroxisomes [26]. We suggested that the increase of Fis1 and Dnm1l in the fetal pancreas was an adaptive response to the potential defect in peroxisomal biogenesis and metabolism caused by deregulated peroxins and Acox.

Several studies have shown that functional peroxisomes are necessary to prevent ER stress. Peroxisome deficiency activates endoplasmic reticulum stress pathways in the liver, mediated by PERK and ATF4 signaling [27, 28]. Our LC-MS results showed an increase in eif4a2 and eif2b2 and a decrease in Dnajc2 in IUGR fetal pancreas. We also found that protein malnutrition triggers an ER stress with PERK and ATF6 signaling activated (in another report) in IUGR fetal pancreas. Our results illustrated that besides disrupted FAO metabolism, the deficient peroxisome also trigged ER stress in IUGR pancreas.

The present study also showed alterations in mitotic cell cycle regulators in the IUGR fetal pancreas, including Mcm2/5, Cdk1, and Brd4. Mcm2 and 5 are components of the Mcm2-7 complex, which plays an important role in the initiation of DNA replication and contributes to replication elongation, condensation, transcription, and recombination of DNA molecules [29]. Mcm2-7 interact with each other to form a functional DNA helicase, which triggers the initial step of DNA synthesis. Each member of the Mcm complex may play a distinct or similar role in the regulation of cell behavior. Moreover, Mcm2 plays a role in ciliogenesis in postmitotic tissues beyond promoting DNA synthesis [30]. Cdk1 is a highly conserved protein that functions as a serine/threonine kinase and is a central regulator that drives cells through the G2 phase and mitosis. Knockout of Cdk1 leads to arrest of embryonic development around the blastocyst stage [31]. Brd4, a member of the bromodomain and extraterminal protein family, plays a positive role in cell growth and cell cycle progression by binding to acetylated histones and transcription factors and recruiting various transcriptional regulators [32]. It can also repress autophagy and lysosomal gene expression [33]. Brd4 inhibition suppresses cell cycle progression and enhances autophagic flux and liposomal function, promoting the degradation of aggregated proteins. We speculated that disorders of cell cycle regulators may induce cell division arrest and inhibit the differentiation and development of islets and cells. The specific interactions between multiple factors and the mechanisms underlying the effects on the developing pancreas remain to be studied with a cell model.

Kelly et al. [34] also reported that cell cycle regulators and the antioxidant were involved in the mechanisms of pancreatic dysplasia in hyperthermia-induced IUGR fetal sheep by RNA sequencing, though specific regulatory factors were different from our results. We speculated that the discrepancy was derived from different models (hyperthermia-induced sheep vs. protein restriction-induced rats), different targets (RNA sequencing vs. proteomics), and different tissue (islets vs. whole pancreas) in these studies. It suggested that mal-environment in utero has a different effect on endocrine and exocrine cells, so separating different cells of the pancreas for study will be more precise, and different aetiologies lead to different changes in the pancreas, so one type of animal model cannot completely simulate the condition of human diseases.

Epigenetic regulation of gene expression via histone acetylation modulates many cellular processes, including apoptosis, cell cycle, and cell growth and differentiation. In mammals, there are 18 Hdac enzymes that are divided into four classes. Among these classes, class I, class II, and class IV Hdacs are zinc-dependent enzymes, whereas class III (sirtuins) is dependent on nicotinamide adenine dinucleotide [35]. In the SIRT protein family (Sirt1–7), Sirt1, Sirt6, and Sirt7 catalyze the posttranslational modification of proteins in the nucleus. Sirt3, Sirt4, and Sirt5 are in the mitochondria, whereas Sirt2 is expressed in the cytosol. Hdac1/2 (class I) proteins were downregulated, whereas Sirt1 and 3 (class III) were upregulated in the IUGR fetal pancreas. Acetylation of histone H3 lysine 56 (H3K56) is regulated by Hdac1/2 [36], whereas Sirt3 also interacts with acetylated H3K56ac [37]. The downregulation of H3K56ac in the present study may be related to the upregulation of Sirt3. Future research will focus on defining *in vivo* targets of Hdacs to provide insight into their effect on the development and metabolism of the pancreas in a deficient intrauterine environment.

The present study is the first to identify differentially expressed proteins in the fetal pancreas of IUGR by proteomic analysis. We found that the dysregulated proteins were involved in peroxisome biogenesis and fission, FAO, mitotic cell cycle, and histone modification. These variations may be responsible for disorders of pancreatic development and metabolism in the IUGR pancreas. The present data provide insight into the role of the peroxisome in pancreatic development and may be valuable for furthering our understanding of the pathogenesis of IUGR-induced diabetes.

## Figures and Tables

**Table 1:** Proteins chosen for validation.

Protein ID	Gene symbol	Proteins	Unique peptides	Average iTRAQ ratio (*I*/*C*)	*p* value	Main function
Q642G4	Peroxisomal membrane protein Pex14	1	1	0.2805	0.011	An essential component of the peroxisomal import machinery. Plays a key role for peroxisome movement through a direct interaction with tubulin.
Q6IN39	Peroxisomal multifunctional enzyme type 2 Hsd17b4	2	6	0.5702	0.05	Bifunctional enzyme acting on the peroxisomal beta-oxidation pathway for fatty acids.
D3ZVU7	Histone deacetylase1	3	3	0.4278	0.0053	Responsible for the deacetylation of lysine residues on the N-terminal part of the core histones (H2A, H2B, H3, and H4).
P39951	Cyclin-dependent kinase 1	19	3	0.5939	0.0223	Plays a key role in the control of the eukaryotic cell cycle by modulating the centrosome cycle as well as mitotic onset
D3ZP96	DNA helicase MCM2	1	3	0.5621	0.0033	A component of the MCM2-7 complex (MCM complex) which is the putative replicative helicase essential for “once per cell cycle” DNA replication initiation and elongation in eukaryotic cells. Plays a role in cell apoptosis.
B2GUX3	DNA helicase MCM5	3	6	2.0485	0.0283	A component of the MCM2-7 complex. Interacts with MCMBP.
Have/no						
D3ZGX8	Bromodomain-containing 4	2	1	None	Chromatin reader protein that recognizes and binds acetylated histones and plays a key role in transmission of epigenetic memory across cell divisions and transcription regulation.	
F1LQB2	Structural maintenance of chromosomes protein Smc3	3	5	None	Central component of cohesin, a complex required for chromosome cohesion during the cell cycle.	
Q08420	Extracellular superoxide dismutase [Cu-Zn]SOD3	1	1	Have	Protect the extracellular space from toxic effect of reactive oxygen intermediates by converting superoxide radicals into hydrogen peroxide and oxygen.	

C: control; I: IUGR; peptide: the number of peptides identified by LC-MS/MS.

## Data Availability

The data used to support the findings of this study are available from the corresponding author upon request.

## Abbreviations

IUGR: Intrauterine growth restriction
T2DM: Type 2 diabetes mellitus
GSIS: Glucose-stimulated insulin secretion
LC-MS: Liquid chromatography-tandem mass spectrometry
BP: Biological process
MF: Molecular function
CC: Cellular component
GO: Gene Ontology
IPA: Ingenuity Pathway Analysis
DEPs: Differentially expressed proteins
IF: Immunofluorescence
SOD: Superoxide dismutase
FAO: Fatty acid beta-oxidation
EIF2: Eukaryotic translation initiation factor 2
ATF4: Activating transcription factor 4
PERK: Protein kinase R-like endoplasmic reticulum kinase
HDAC: Histone deacetylase
ERS: Endoplasmic reticulum stress
ACOX: Acyl-CoA oxidase
DBP: D-bifunctional protein
CDK1: Cyclin-dependent kinase 1
BRD4: Bromodomain-containing protein 4.

## Supplementary Materials

Supplementary Materials Supplement table 1: antibodies for western blotting and IHC. Supplement table 2: primers for Q-PCR.
