# Supplementary material for: Label-Free Proteomics of the Fetal Pancreas Identifies Deficits in the Peroxisome in Rats with Intrauterine Growth Restriction
Source: Oxid Med Cell Longev. 2019 Nov 3;2019:1520753. doi: 10.1155/2019/1520753 (PMC6874927; doi:10.1155/2019/1520753)
Supplement: Supplementary Materials — Supplement table 1: antibodies for western blotting and IHC. Supplement table 2: primers for Q-PCR. [file 1520753.f1.docx]

**Supplement table1. antibodies for western blotting and IHC**

| Target  protein | Source | Company | Ref. | MW (kDa) |
| --- | --- | --- | --- | --- |
| Pex14 | Rabbit | Enogene | E2A4284 | 38 |
| PEX19 | Rabbit | Enogene | E2A7361 | 40 |
| PEX3 | Rabbit | Enogene | E2A4282 | 45 |
| Pex11b | Rabbit | Enogene | E2A4283 | 28 |
| PMP70 | Rabbit | Immunoway | YN0479 | 72 |
| Hsd17b4 | Rabbit | Immunoway | YT5386 | 80 |
| CDK1 | Rabbit | Proteintech | 19532-1-AP | 34 |
| MCM2 | Rabbit | Enogene | E1A0206 | 120 |
| MCM5 | Rabbit | Enogene | E1A0207 | 90 |
| Brd4 | Rabbit | Abcam | Ab128874 | 152 |
| Smc3 | Rabbit | Proteintech | 14185-1-AP | 140 |
| HDAC1 | Rabbit | Immunoway | YT5386 | 80 |
| HDAC2 | Rabbit | Proteintech | 12922-3-AP | 55 |
| Sirt1 | Rabbit | Proteintech | 13161-1-AP | 100 |
| Sirt2 | Rabbit | Enogene | E1A5256 | 43 |
| Sirt3 | Rabbit | Enogene | E1A5135 | 29 |
| β-actin | mouse | Proteintech | 60008-1-lg | 43 |

**Supplement table2. primers for Q-PCR**

| **Gene name** | **Accession**  **Number** | **Primer Sequences(**5’ -3’) | **Size**  **(bp)** | **Annealing temp (℃)** |
| --- | --- | --- | --- | --- |
| ***Pex14*** | NM_001013911 | GTACAGAGGGAGGACAGACG  TCTCGCTCACTCTCATTGCT | 98 | 60 |
| ***Pex11b*** | NM_001025684 | TTTAGTGCTCAGAGCCAAGC  GCTCAGATGACCCTCCAGTT | 141 | 60 |
| ***Pex19*** | NM_001107375 | CAATGAAGGAGCTGGCTGAG  CTGCGTTCTGAAGGTCAGTG | 171 | 60 |
| ***Acox2*** | [NM_145770](https://www.ncbi.nlm.nih.gov/nucleotide/NM_145770.2?report=genbank&log$=nuclalign&blast_rank=4&RID=DPN74MZU015) | GCCTTACACGGTGTTCTGAC  CATCCTTCCGGATCAAGGGA | 121 | 60 |
| ***Acox3*** | [NM_053339](https://www.ncbi.nlm.nih.gov/nucleotide/NM_053339.1?report=genbank&log$=nuclalign&blast_rank=24&RID=DPN9WC8S01R) | CTAGCCATGAACCGGTTTGG  TGTGAAGTGAGCTCCGTCTT | 147 | 60 |
| ***Hsd17b4*** | NM_024392 | ATGCCAGAAGACCTCGTTGA  TGCTCCAACCTCAAACAAGC | 111 | 60 |
| ***Hsd17b13*** | [NM_001009684](javascript:if(window.name=='')%20%7b%20window.location.href='./nil';%20%7d%20else%20%7b%20dynPopitupType('NCBI_REFSEQ__TRANSCRIPT',%20'http://www.ncbi.nlm.nih.gov/entrez/query.fcgi?db=nucleotide&term=NM_001009684');%20%7d) | AGGAGCTGGACACGGAATAG  TCCTCAACACCGTGCTTACT | 99 | 60 |
| ***Brd4*** | NM_001191934 | AGTCCCGGGAGTACAGAGAT  AACTGGCTCTTCAGGCTCAT | 173 | 60 |
| ***CDK1*** | NM_019296 | ATGGCCCTTAAGCACCCATA  CCAACAGTAAACGCCACGAT | 112 | 60 |
| ***Smc3*** | NM_031583 | TACAGGAAAGCAAGGCGAGA  GCATCCAAAGCTTGGTCGAT | 147 | 60 |
| ***Mcm5*** | NM_001106170 | ACAAGTGCAAGTGTGTGGAC  CAATACAGCTGCATGTGCCT | 97 | 60 |
| ***Mcm2*** | NM_001107873 | TCAACCAGATGGACCAGGAC  TGATGGGAATACTGCCCGTT | 84 | 60 |
| ***Hdac1*** | NM_001025409 | CCTCACCGAATCCGAATG  CGAATAGAACGCAAGAACTTG | 164 | 60 |
| ***Sirt1*** | [XM_017588054](https://www.ncbi.nlm.nih.gov/nucleotide/XM_017588054.1?report=genbank&log$=nuclalign&blast_rank=7&RID=DPP3YP8Y014) | TTTCAGAACCACCAAAGCG  TCCCACAGGAAACAGAAACC | 126 | 60 |
| ***Sirt3*** | [NM_001106313](https://www.ncbi.nlm.nih.gov/nucleotide/NM_001106313.2?report=genbank&log$=nuclalign&blast_rank=31&RID=DPR4W0X9014) | ACAAGAGCTGCAGGATCTCA  CGCAGGTGAAGAAGTAAGCC | 91 | 60 |
| ***β-actin*** | NM_031144 | AGTCCCTCACCCTCCCAAAAG  AAGCAATGCTGTCACCTTCCC | 96 | 60 |
